# Characterization of the hot pepper (*Capsicum frutescens*) fruit ripening regulated by ethylene and ABA

**DOI:** 10.1186/s12870-018-1377-3

**Published:** 2018-08-10

**Authors:** Bing-Zhu Hou, Chun-Li Li, Ying-Yan Han, Yuan-Yue Shen

**Affiliations:** College of Plant Science and Technology, Beijing Key Laboratory for Agricultural Application and New Technique, Beijing University of Agriculture, Beijing, 102206 China

**Keywords:** Hot pepper (*Capsicum frutescens*), Fruit ripening, Carotenoid biosynthesis, Chlorophyll degradation, Ethylene, Abscisic acid

## Abstract

**Background:**

Ripening of fleshy fruits has been classically defined as climacteric or non-climacteric. Both types of ripening are controlled by plant hormones, notably by ethylene in climacteric ripening and by abscisic acid (ABA) in non-climacteric ripening. In pepper (*Capsicum*), fruit ripening has been widely classified as non-climacteric, but the ripening of the hot pepper fruit appears to be climacteric. To date, how to regulate the hot pepper fruit ripening through ethylene and ABA remains unclear.

**Results:**

Here, we examined ripening of the hot pepper (*Capsicum frutescens*) fruit during large green (LG), initial colouring (IC), brown (Br), and full red (FR) stages. We found a peak of ethylene emission at the IC stage, followed by a peak respiratory quotient at the Br stage. By contrast, ABA levels increased slowly before the Br stage, then increased sharply and reached a maximum level at the FR stage. Exogenous ethylene promoted colouration, but exogenous ABA did not. Unexpectedly, fluridone, an inhibitor of ABA biosynthesis, promoted colouration. RNA-sequencing data obtained from the four stages around ripening showed that *ACO3* and *NCED1*/*3* gene expression determined ethylene and ABA levels, respectively. Downregulation of *ACO3* and *NCED1*/*3* expression by virus-induced gene silencing (VIGS) inhibited and promoted colouration, respectively, as evidenced by changes in carotenoid, ABA, and ethylene levels, as well as carotenoid biosynthesis-related gene expression. Importantly, the retarded colouration in *ACO3*-VIGS fruits was rescued by exogenous ethylene.

**Conclusions:**

Ethylene positively regulates the hot pepper fruit colouration, while inhibition of ABA biosynthesis promotes colouration, suggesting a role of ABA in de-greening. Our findings provide new insights into processes of fleshy fruit ripening regulated by ABA and ethylene, focusing on ethylene in carotenoid biosynthesis and ABA in chlorophyll degradation.

**Electronic supplementary material:**

The online version of this article (10.1186/s12870-018-1377-3) contains supplementary material, which is available to authorized users.

## Background

Peppers (*Capsicum*), as the most widely cultivated vegetable crop, play important roles in human food, nutrition, and health [1]. The *Capsicum* genus includes five species: *C. annuum*, *C. chinense*, *C. frutescens*, *C. pubescens*, and *C. baccatum*, which are known as bell, chili, jalapeno, cayenne, and cherry types, respectively [2–4]. Generally, bell peppers (*C. annuum*) are harvested at the horticulturally mature green stage for fresh food, while hot peppers (*C. frutescens*) are harvested at the ripening stage for processing or drying. The pepper fruit ripening has been typically classified as non-climacteric [5–7]. However, given the wide diversity of peppers, patterns of ethylene production and respiratory rates vary with pepper species, and the hot pepper fruit ripening appears to be climacteric [8]. To our knowledge, how to regulate the hot pepper fruit ripening by ethylene and ABA remains unclear.

Ripening of fleshy fruits is typically classified as climacteric or non-climacteric based on ethylene emission and respiration during ripening [9–11]. During ripening, climacteric fruits show a burst in ethylene emission and respiration, whereas no peak in ethylene emission occurs in non-climacteric fruits [11, 12]. Based on various tomato (*Solanum lycopersicum*) mutants, including *never-ripe* (*nr*), *colourless non-ripening* (*cnr*), *ripening inhibitor* (*rin*), and *non-ripening* (*nor*) *mutants*, the molecular mechanisms of climacteric fruit ripening have been defined by ethylene biosynthesis and signalling [13, 14]. Ethylene biosynthesis is controlled by two key steps which are catalysed sequentially by 1-aminocyclopropane-1-carboxylic acid (ACC) synthases (ACSs) and ACC oxidases (ACOs) [15]. Ethylene is perceived by a family of five ethylene receptors (ETR1, ETR2, ERS1, ERS2, and EIN4) which interact with a downstream constitutive triple response 1 (CTR1) kinase, which activates ethylene insensitive 2 (EIN2) by a negative mechanism and in turn evokes a transcriptional cascade involving EIN3/ EIN3-like (EIL) and ethylene responsive factors [16–20]. A coordinated regulation between ethylene biosynthesis and ethylene signalling by the tomato MADS-box protein, LeMADS-RIN, is required for ripening [21].

Although the mechanisms of non-climacteric fruit ripening regulated by plant hormones are not fully understood [14, 19], increasing evidence indicates an important role of ABA in ripening [22–29]. Tobacco rattle virus (TRV)-induced gene silencing (VIGS) in strawberry fruit, a model of non-climacteric ripening, showed that downregulation of the mRNA expression levels of *FaNCED1* or *FaBG3*, key to ABA biosynthesis, led to a significant reduction in ABA contents and then inhibition of ripening, respectively [23, 24]. Similarly, downregulation of the mRNA expression levels of *FaPYR1*/*FaABAR* and *FaABI1*/*FaSnRK2.6*, key to ABA signalling, affected ripening [22, 23, 27]. FaMYB10 is a signal transduction mediator to play a role in ABAR (putative ABA receptor)-mediated anthocyanin synthesis during strawberry fruit ripening [28]. Vital roles for hormones in fleshy fruit ripening are now accepted, particularly for ethylene in climacteric fruit and for ABA in non-climacteric fruit [12, 14, 19, 20, 30, 31].

Notably, the ripening of some non-climacteric fruits in guava, melon, Japanese plum, Asian pear, and hot pepper, is climacteric [32, 33]. Pepper fruit ripening is typically considered to be non-climacteric [5–7, 34–38], while some hot pepper fruit ripening is climacteric, and patterns of ethylene production and respiratory rates differ with pepper species [8, 39]. Thus, characterisation of hot pepper fruit ripening regulated by ABA and ethylene contributes to understanding of fleshy fruit ripening.

In this study, we used a red, clustered, upright, bullet-shaped and hot pepper (*Capsicum frutescens* cv. ‘Chaotianjiao 6’) to examine fruit ripening during large green (LG), initial colouring (IC), brown (Br), and full red (FR) stages. We performed a series of physiological, pharmacological [ethylene and its inhibitor 1-methylcyclopropene (1-MCP), and ABA and its inhibitor, fluridone], RNA-sequencing (RNA-seq), and VIGS experiments to assess the roles of ethylene and ABA in the hot pepper fruit ripening. We found that ABA and ethylene regulate hot pepper ripening differently, showing either climacteric or non-climacteric characteristics.

## Results

### Morphological and physiological changes at the onset of pepper fruit ripening

**Fig. 1:**
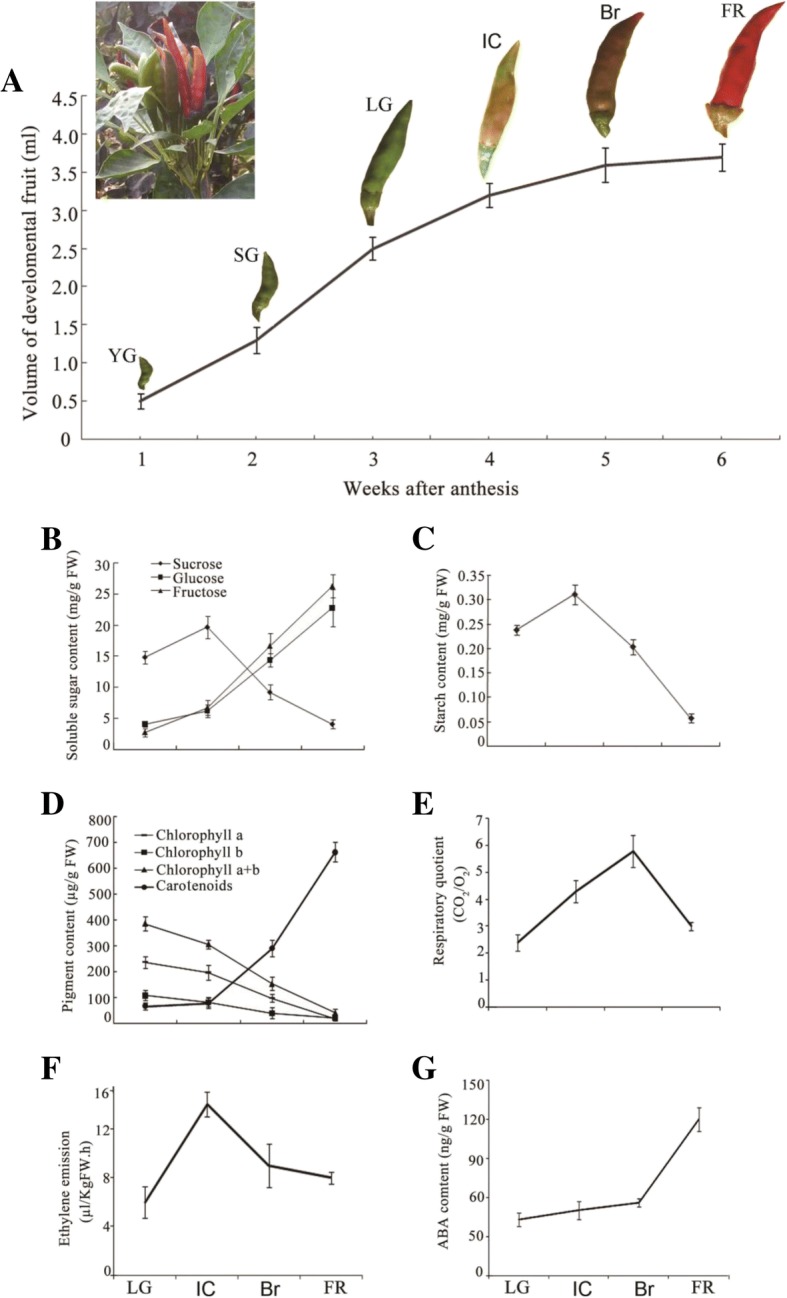
Morphological and physiological variation in pericarp tissues during pepper fruit ripening. The developmental pepper fruit was divided into six stages – young green (YG), small green (SG), large green (LG), initial colouring (IC), brown (Br), and full red (FR) – at 1, 2, 3, 4, 5, and 6 weeks after fruit set, respectively. Pericarp tissues were used for physiological analysis. Changes in (**a**) size and colour during fruit development, (**b**) soluble sugar (sucrose, glucose, and fructose) content, (**c**) starch content, (**d**) pigment content, (**e**) respiratory quotient, (**f**) ethylene emission, and (**g**) ABA content. Error bars represent the standard error (SE, *n* = 3)

We divided the development of ‘Chaotianjiao 6’ (Additional file 1: Figure S1) pepper fruit into six stages: young green (YG), small green (SG), large green (LG), initial colouring (IC), brown (Br), and full red (FR) at 1, 2, 3, 4, 5, and 6 weeks after anthesis, respectively, of which the later four stages corresponded to the onset of ripening (Fig. 1a). Concurrent with the onset of ripening, glucose, fructose, and carotenoid contents increased rapidly (Fig. 1b, d), while sucrose and starch contents declined sharply after the IC stage (Fig. 1b, c) and chlorophyll contents decreased gradually (Fig. 1d). Notably, ethylene emission reached a peak at the IC stage, concurrent with changes in colouration (Fig. 1f), followed by a peak in the respiratory quotient (RQ) at the Br stage (Fig. 1e). Interestingly, the drastic reduction in the RQ (Fig. 1e) was accompanied by rapid accumulation of ABA after the Br stage (Fig. 1g). These results suggested that both ethylene and ABA might be involved in ripening.

### Effects of ABA and ethylene and their inhibitors on pepper fruit ripening

**Fig. 2:**
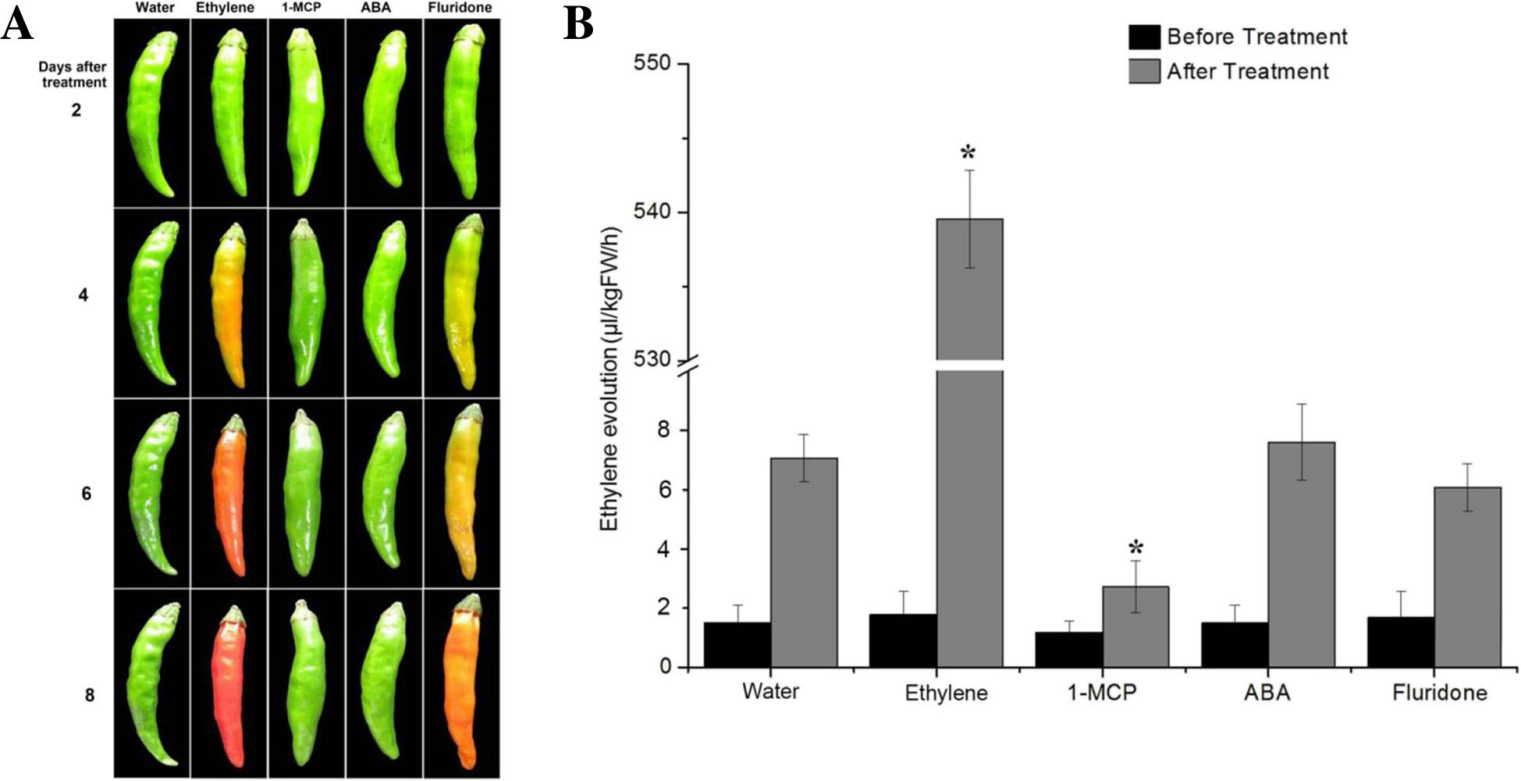
Effects of ABA, ethylene, and inhibitors on pepper fruit ripening. Twenty one LG fruits attached to plants were used for treatment (*n* = 7, three replications) and water was used as a control. The treated and control fruits were placed in a growth chamber for 8 days. Three treated fruits were used to determine ethylene emission levels. (**a**) Fruits were treated with 50 μM ABA, 100 μM fluridone, 50 μM ethephon, or 1 mL/L 1-MCP. (**b**) Ethylene emission in the ABA-, fluridone-, ethylene-, and 1-MCP-treated fruits. Error bars represent the SE (n = 3). An asterisk (*) in columns of the same colour indicates a statistically significant difference (*P* < 0.05) based on analysis of variance followed by Duncan’s multiple-range test

It is previously reported that detached pepper fruits are suitable for the study of ripening [8]. Therefore, we used detached LG-stage pepper fruits to investigate the effects of ABA and ethylene on ripening. LG fruits were treated for 8 days with 50 μM ABA, 100 μM fluridone, 50 μM ethephon, or 1 mL/L 1-MCP (*n* = 7, three replications). Relative to the control treatment with water, colouration was promoted by ethephon and fluridone (21 FR fruits and 21 orange fruits, respectively), whereas the ABA and 1-MCP treatments had no effect on colouration (Fig. 2a). Ethylene emission was significantly promoted and inhibited by the ethephon and 1-MCP treatments, respectively, while was not affected significantly by the ABA and fluridone treatments (Fig. 2b). However, the endogenous ABA levels were significantly affected by the ABA and fluridone treatments, resulting in endogenous ABA levels of 207 ± 12 and 34 ± 2.1 μg/g fresh weight (FW), respectively. These results suggest that ethylene plays a role in the regulation of ripening, and that the role of ABA is complex.

### RNA-seq analysis of gene expression during the onset of pepper fruit ripening

**Fig. 3:**
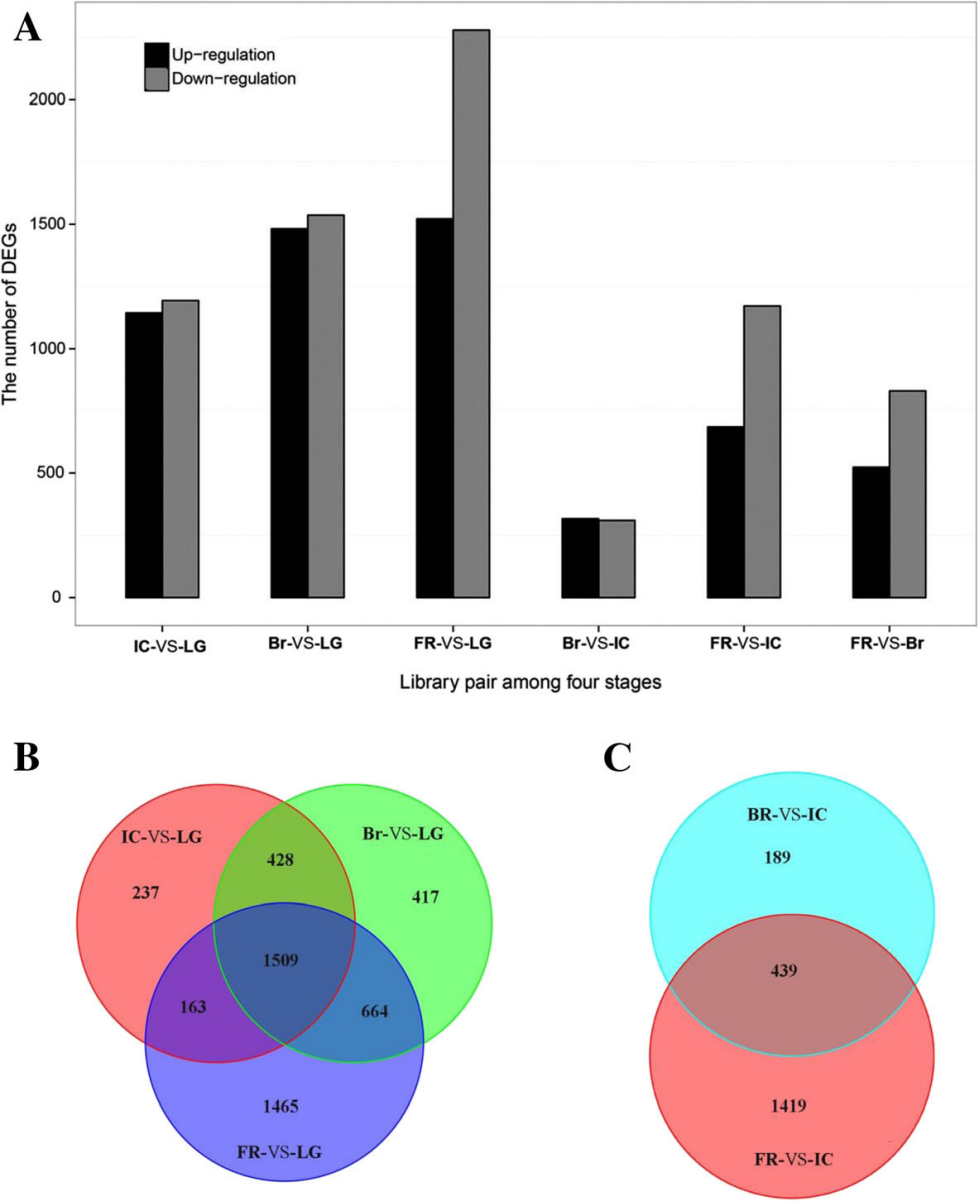
Differentially expressed genes (DEGs) between each pair of ripening stage cDNA libraries. Four cDNA libraries corresponded to the LG, IC, Br, and FR fruit ripening stages. (**a**) Numbers of up- and down-regulated DEGs between library pairs. (**b**) Venn diagram comparing the DEGs between the IC, Br, and FR libraries and the LG library. (**c**) Venn diagram comparing the DEGs between the Br and FR libraries and the IC library

**Fig. 4:**
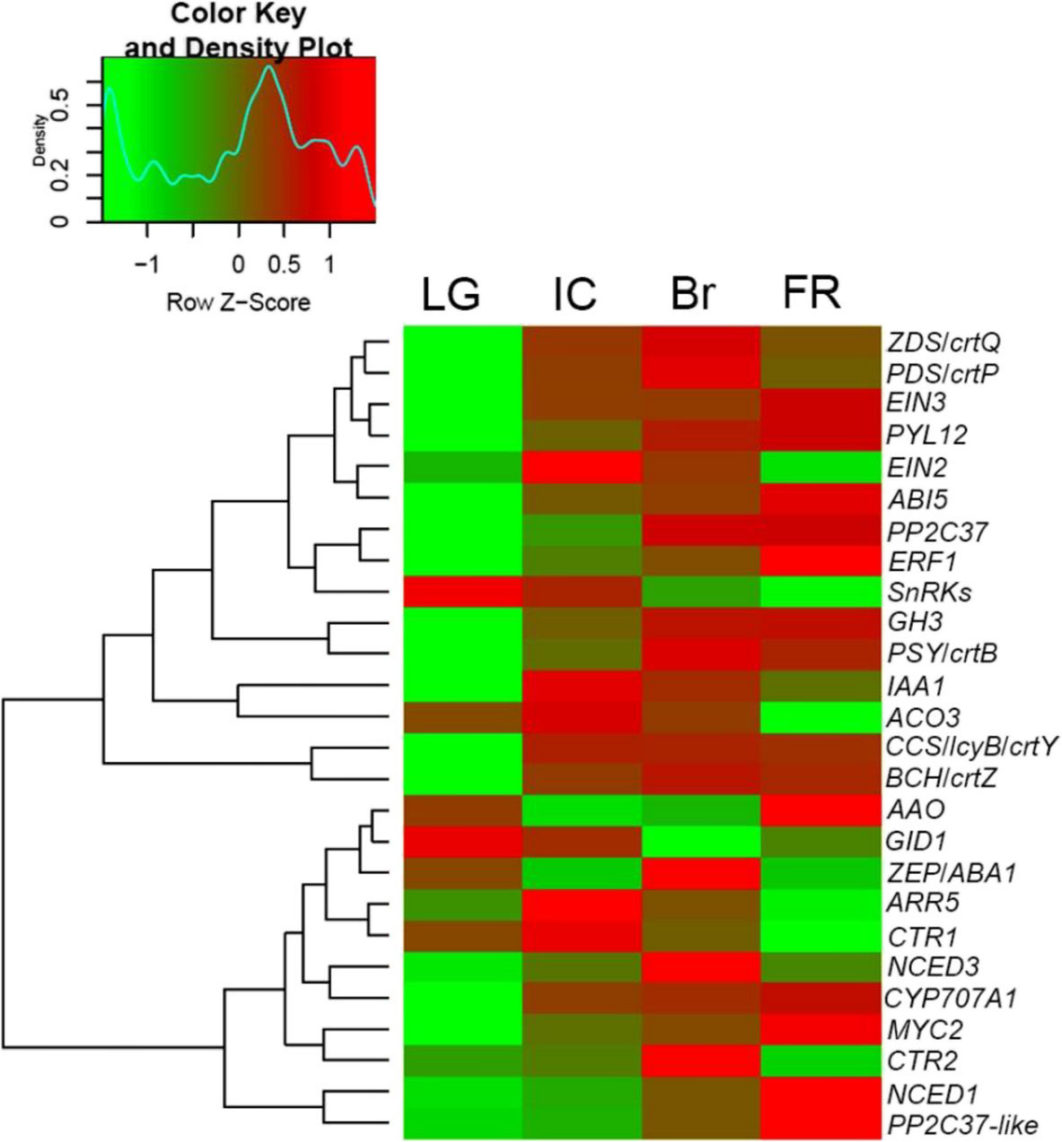
Heat map and cluster analysis of transcripts of genes related to ripening. Gene expression level data were normalised to z-scores using the formula log_10_ (FPKM + 1) by colour key and density plot. The transition from green to red represents the value of gene expression from low to high during fruit ripening. The genes annotated in relevant pathways: carotenoid biosynthesis (PSY/crtB: 15-cis-phytoene synthase; PDS/crtP: 15-cis-phytoene desaturase; ZDS/crtQ: zeta-carotene desaturase; BCH/crtZ1: beta-carotene hydroxylase 1; CCS/lcyB/crtY: capsanthin/capsorubin synthase); ABA biosynthesis and metabolism (ZEP/ ABA1: zeaxanthin epoxidase; NCED1: 9-cis-epoxycarotenoid dioxygenase1; NCED3: 9-cis-epoxycarotenoid dioxygenase 3; AAO1: abscisic-aldehyde oxidase 1; CYP707A1, abscisic acid 8′-hydroxylase); ABA signaling (PYL12: abscisic acid receptor; PP2C37: protein phosphatase 2C 37; PP2C37-like: protein phosphatase 2C 37-like; SnRK2: serine/threonine-protein kinase SRK2-like; ABI5: abscisic acid-insensitive 5-like protein); ethylene biosynthesis and signaling (ACO3: 1-aminocyclopropane-1-carboxylate oxidase 3; CTR1: ethylene response sensor 1; CTR2: ethylene response sensor 2; EIN2: ethylene-insensitive protein 2; EIN3: ethylene-insensitive protein 3; ERF1: ethylene-responsive transcription factor 1B-like); IAA biosynthesis and signaling (GH3: indole-3-acetic acid-amido synthetase GH3.1; IAA, auxin-responsive protein IAA); GA signaling (GID1,gibberellin receptor GID1); JA signaling (MYC2, transcription factor MYC2); cytokinin signaling (ARR5: two-component response regulator 5)

To understand the molecular mechanisms by which plant hormones regulate pepper fruit ripening, we performed RNA-seq analysis of cDNA libraries corresponding to four stages during the onset of pepper fruit ripening. Genetic databases used to annotate unigenes identified by RNA-seq analysis including the National Center for Biotechnology Information non-redundant protein database, SWISS-PROT, TrEMBL, Cdd, Pfam, the KOG database, the Kyoto Encyclopedia of Genes and Genomes, and Gene Ontology based on an E-value of 1e^− 5^ and identity of 30%. Differentially expressed genes (DEGs) were identified between the LG and IC stages as 2337 genes with 1145 upregulated and 1192 downregulated, the LG and Br stages as 3018 genes with 1482 upregulated and 1536 downregulated, the LG and FR stages as 3801 genes with 1522 upregulated and 2279 downregulated, the IC and Br stages as 628 genes with 317 upregulated and 311 downregulated, the IC and FR stages as 1858 genes with 686 upregulated and 1172 downregulated, and the Br and FR stages as 1354 genes with 524 upregulated and 830 downregulated (Fig. 3a). Some of these DEGS were specific to particular pairs of ripening stages; 237 DEGs were specific to LG/IC, 417 DEGs were specific to LG/Br, 1464 DEGs were specific to LG/FR (Fig. 3b), 189 DEGs were specific to IC/Br, and 1419 DEGs were specific to IC/FR (Fig. 3c). Among these DEGs, we identified genes related to phytohormone and carotenoid pathways, including carotenoid biosynthesis genes (*PSY*/*crtB*, *PDS*/*crtP*, *ZEP*, *ZDS*/*crtQ*, *BCH*/*crtZ1*, and *CCS*/*lcyB*/*crtY*), ABA biosynthesis and metabolism genes (*ABA1*/*ZEP*, *NCED1*, *NCED3*, *AAO1*, and *CYP707A1*), ABA signalling genes (*PYL12*, *PP2C37*, *PP2C37-like*, *SnRK2*, and *ABI5*), ethylene biosynthesis and signalling genes (*ACO3*, *CTR1*, *CTR2*, *EIN2*, *EIN3*, and *ERF1*), auxin biosynthesis and signalling genes (*GH3* and *IAA*), a GA (gibberelin) signalling gene (*GID1*), a JA (jasmonic acid) signalling gene (*MYC2*), and a cytokinin signalling gene (*ARR5*; Fig. 4).

Consistent with the changes in fruit colouration during ripening, the DEGs with continuously decreasing transcript levels included *SnRK2* and *GID1*, and the DEGs with continuously increasing transcripts included *EIN3*, *ERF2*, *ABI5*, *PP2C37*, *NCED1*, *CYP707A1*, *AAO*, *GH3*, and *MYC2*. The DEGs with highest transcript levels at the IC stage included *EIN2*, *ACO3*, *CTR1*, *IAA1*, and *ARR5*, and the DEGs with highest transcript levels at the Br stage included *CTR2*, *NCED3*, *ZDS*, *PDS*, *crtB*, *crtZ1*, and *ZEP* (Fig. 4). Importantly, ABA-related genes (*n* = 10) constituted the largest portion of the phytohormone-related DEGs during ripening, followed by ethylene-related genes (*n* = 6), and only two DEGs related to other phytohormones were identified. Interestingly, transcription of the ethylene-related DEGs during ripening showed a coordinated expression pattern between ethylene biosynthesis and signalling, whereas transcripts of the ABA-related DEGs showed no coordinated expression patterns between ABA biosynthesis and signalling (Fig. 4). More importantly, *ACO3* transcript levels exhibited a trend similar to ethylene levels, whereas *NCED1* and *NCED3* transcript levels, in particular *NCED1*, exhibited trends consistent with ABA contents (Figs. 1, 4). Taken together, the transcriptome data related to phytohormone-pathway DEGs suggest that ethylene plays an important role in ripening, and that the role of ABA is complex.

### VIGS of key genes to ABA and ethylene biosynthesis during pepper fruit ripening

**Fig. 5:**
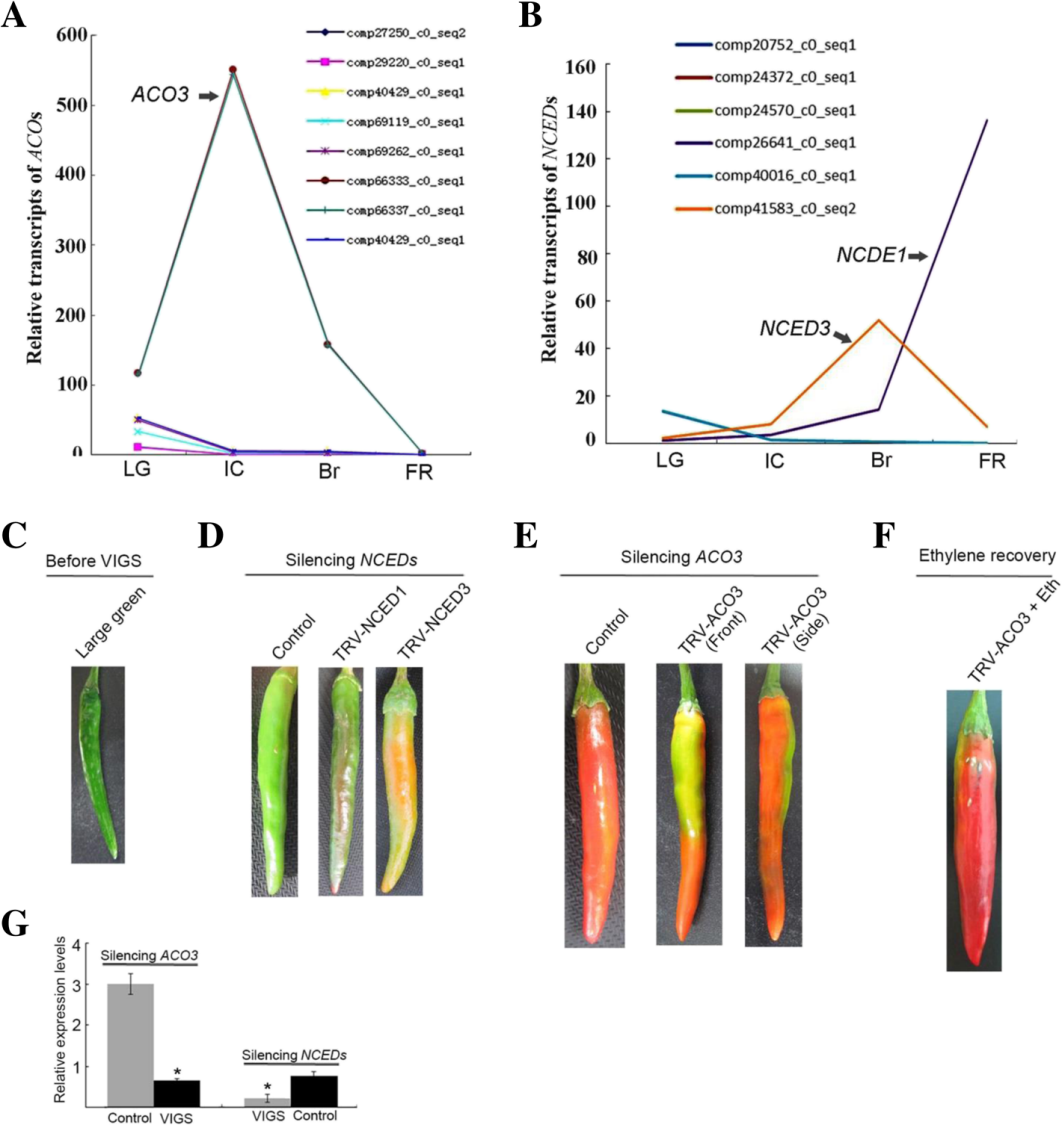
Phenotypes of *NCEDs*-VIGS and *ACO3*-VIGS transgenic fruits. Four ACO gene family members (**a**) and three *NCED1*/*3* gene family members (**b**) were identified based on the log_2_ gene expression levels obtained from RNA-seq data during fruit ripening, including the LG, IC, Br, and FR stages. (**c**) Nineteen-day-old LG fruit attached to the plants were used for VIGS. (**d**) About 1 week after infiltration, the *NCED1*/*3*-VIGS fruits exhibited colour development, while the control fruits infiltrated with TRV alone remained in the LG stage. (**e**) Three weeks after infiltration, the *ACO3*-VIGS fruits exhibited a chimeric phenotype, while the control fruits infiltrated with TRV alone had reached the FR stage. (**f**) Exogenous 50 μM ethephon rescued the chimeras, with ripening to the FR stage. (**g**) *ACO3* mRNA expression in VIGS fruits was downregulated more than 80% relative to the control, and the average levels of *NCED1* and *NCED3* expression in VIGS fruits were downregulated more than 70% relative to the control. *Ubi3* expression served as the internal control. Error bars represent the SE (n = 3). An asterisk (*) in the columns with same colour indicates a statistically significant difference (*P* < 0.05) based on analysis of variance followed by Duncan’s multiple-range test

To confirm the roles of *ACO3*, *NCED1*, and *NCED3* in ABA and ethylene biosynthesis, we examined the RNA-seq data for the presence of transcripts of the *ACO* and *NCED* gene families at the onset of ripening. We found that of the four *ACO* gene family members, *ACO3* might play a key role in ethylene biosynthesis (Fig. 5a), and that of the three *NCED* gene family members, *NCED1* and *NCED3* might be important in ABA biosynthesis (Fig. 5b).

To determine the roles of ABA and ethylene in pepper fruit ripening, we used TRV-mediated VIGS [40] to downregulate the mRNA expression levels of *ACO3*, *NCED1*, and *NCED3* during pepper fruit ripening. A mixture of *Agrobacterium* strain GV3101 cultures containing the pTRV1 vector and the pTRV2 vector carrying a 567-bp *ACO3*, a 535-bp *NCED1*, or a 602-bp *NCED3* gene fragment in a 1:1 ratio were syringe-infiltrated into LG fruits attached to the plants (*n* = 7, three replications), and the control fruits were infiltrated with TRV vectors alone (Fig. 5c). Six days after infiltration, most *NCED1*/*3*-VIGS fruits had initiated colouration (18 IC fruits, 3 LG fruits), while all control fruits remained light-green in colour (21 LG fruits; Fig. 5d), demonstrating that the downregulation of *NCED1*/*3* expression promoted fruit colouration. In contrast, 3 weeks after infiltration, all control fruits infiltrated with empty TRV vectors had turned fully red, while the inoculated *ACO3*-VIGS fruits exhibited chimeric phenotypes (21 chimeras; Fig. 5e), demonstrating that the downregulation of *ACO3* expression inhibited fruit colouration. Importantly, these *ACO3*-VIGS chimeric fruits were rescued by exogenous application of 50 μM ethephon 5 days after treatment (Fig. 5f).

To confirm suppression of *ACO3* and *NCED1*/*3* gene expression by VIGS at molecular levels, we performed quantitative polymerase chain reaction (qPCR) analysis. The *ACO3* mRNA level was downregulated in VIGS fruits more than 80% relative to the control, and the average mRNA levels of *NCED1* and *NCED3* in VIGS fruits were downregulated more than 70% relative to the control (Fig. 5g). The combination of transgenic phenotypes and molecular analysis demonstrated that ACO3 positively regulates colouration, whereas NCED1/3 negatively regulates colouration.

### Characterisation of colouration in transgenic pepper fruits at the physiological and molecular levels

**Fig. 6:**
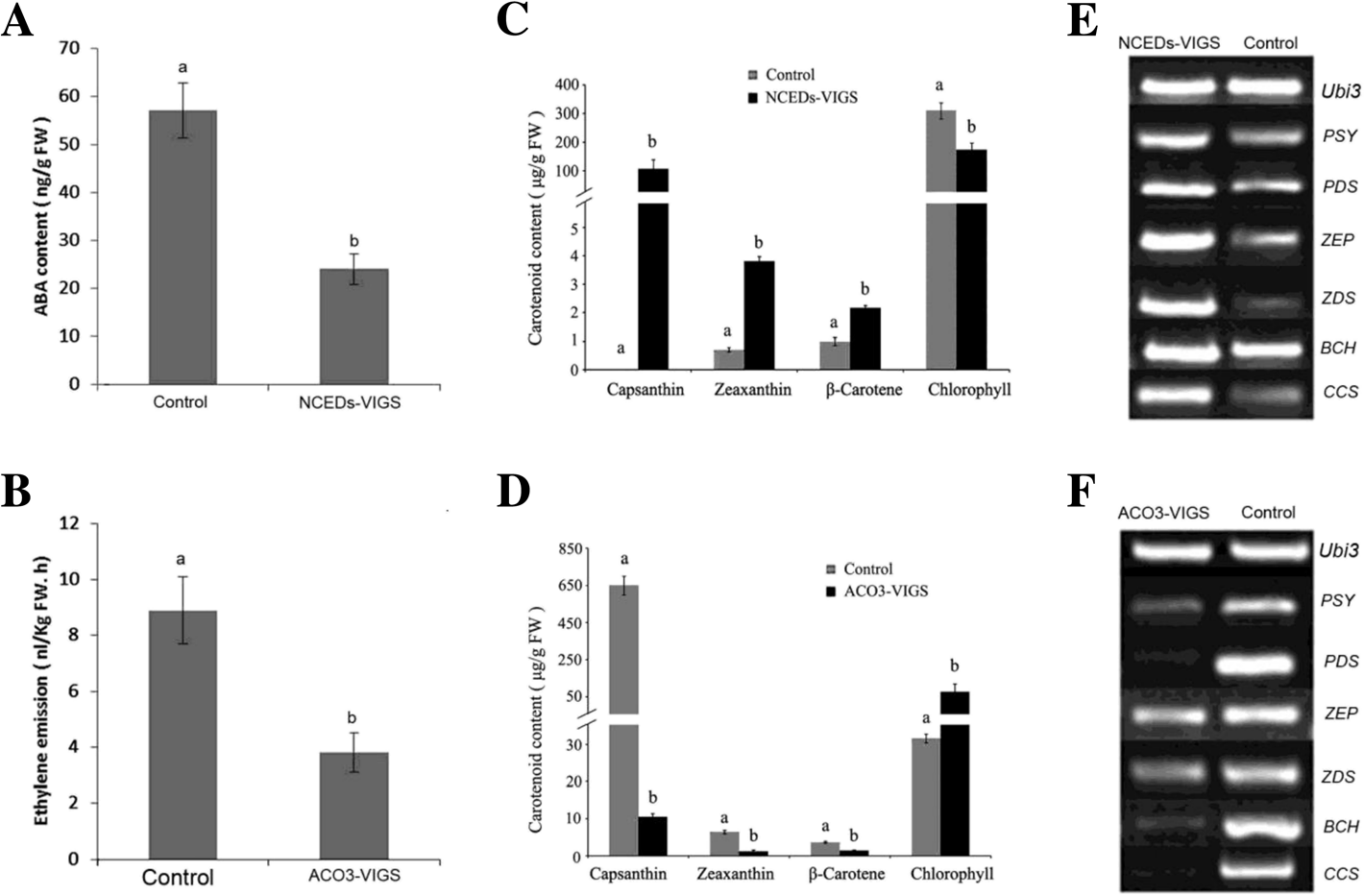
Changes in several physiological and molecular parameters of transgenic and control fruits. Transgenic fruits, in which *NCED* and *ACO3* transcripts were downregulated by more than 70 and 80% relative to control fruits, respectively, were examined. *NCEDs*-VIGS represents *NCED1*-VIGS and *NCED3*-VIGS mixed samples. (**a**) ABA contents. (**b**) Ethylene emission. (**c**) and (**d**) Chlorophyll and carotenoid contents. (**e**) and (**f**) Transcript levels of the *PSY*, *PDS*, *ZEP*, *ZDS*, *BCH*, and *CCS* carotenoid biosynthesis genes. PSY: 15-cis-phytoene synthase; PDS: 15-cis-phytoene desaturase; ZEP: zeaxanthin epoxidase; ZDS: zeta-carotene desaturase; BCH: beta-carotene hydroxylase 1; CCS: capsanthin/capsorubin synthase). *Ubi3* was used as the internal control. Error bars represent the SE (*n* = 3). Columns with different letters (**a**, **b**) indicate statistically significant difference (*p* < 0.05) when the data are performed by variance analysis followed by Duncan’s multiple range tests

To understand colouration in the transgenic pepper fruits, we examined a set of physiological and molecular parameters in VIGS fruits in which *NCED* (mixed samples) and *ACO3* transcripts were downregulated more than 70 and 80%, respectively, relative to their corresponding controls. The measured parameters included ethylene emission, ABA content, pigment (chlorophyll, ß-carotene, zeaxanthin, and capsanthin) concentrations, and transcript levels of carotenoid biosynthesis-related DEGs (*PSY*, *PDS*, *ZDS*, *ZEP*, *BCH*, and *CCS*). ABA content and ethylene emission were significantly reduced in the *NCEDs*-VIGS and *ACO3*-VIGS fruits, respectively (Fig. 6a, b). The downregulation of *NCED* expression promoted capsanthin, zeaxanthin, and ß-carotene accumulation, but decreased the chlorophyll content (Fig. 6c), finally accelerated colouration. In contrast, the downregulation of *ACO3* expression inhibited capsanthin, zeaxanthin, and ß-carotene accumulation as well as chlorophyll degradation (Fig. 6d), which retarded colouration. The promotion and inhibition of colouration in the *NCEDs*-VIGS and *ACO3*-VIGS fruits, respectively, were consistent with the changes in transcript levels of carotenoid biosynthesis-related genes, such as *PSY*, *PDS*, *ZDS*, *ZEP*, *BCH*, and *CCS* (Fig. 6e, f). Our results demonstrated that the downregulation of *NCED* expression inhibited ABA synthesis, finally promoted carotenoid biosynthesis-related gene expression, carotenoid accumulation, and ripening. By contrast, the downregulation of *ACO3* expression inhibited ethylene synthesis, finally inhibiting carotenoid biosynthesis-related gene expression, carotenoid accumulation, and ripening.

## Discussion

During ripening, pepper (*Capsicum*) fruits display a range of colours, including green, yellow, orange, brown, and red, which are due to differences in carotenoid composition and amount [41, 42]. Generally, degradation of chlorophyll together with development of colour is associated with ripening [43]. Pepper fruits with both chlorophyll and red carotenoids appear brown [41]. Interestingly, both climacteric tomato fruit and non-climacteric strawberry fruit exhibit a white stage coupled with chlorophyll degradation during ripening [25, 44]. A recent study showed that the white fruit is a distinct developmental stage and represents the onset of fruit ripening in strawberry [45]. In this study, the colouration by carotenoids in a green background resulted in a brown colour in ‘Chaotianjiao 6’ pepper fruit, which does not have a white stage (Fig. 1). This phenomenon suggests the presence of a ripening mechanism in hot pepper fruit that is distinct from that of tomato or strawberry fruit.

It is well accepted that climacteric fruit ripening shows peaks in ethylene production and respiration, whereas non-climacteric fruit ripening exhibits no significant increase in ethylene production or respiration [46]. Pepper fruit ripening has been widely classified as non-climacteric [35–37, 46, 47]. Nevertheless, the hot pepper fruit ripening exhibits climacteric characteristics [39]. The ripening of some peppers (‘Camelot’, ‘King Arthur’, and ‘Tabasco’) exhibits characteristics intermediate between climacteric and non-climacteric [48]. Interestingly, during ‘Bukang’ pepper fruit ripening, expression of genes involved in ethylene biosynthesis, such as *ACS* and *ACO*, is not induced, while genes encoding ethylene signalling components, such as the *EIL*-like gene, are induced [49]. Although tomato and pepper belong to the Solanaceae family and accumulate carotenoids during ripening, and tomato is used as a model system of climacteric fruit ripening, pepper fruits exhibit diverse ripening types.

*Capsicum frutescens* fruits are generally small, hot, upright, spindle shaped, and red at the ripening stage [50]. In this study, we used the hot, upright, clustered, bullet-shaped, red pepper (*C. frutescens* cv. ‘Chaotianjiao 6’) fruit to assess the regulation of ripening by ethylene and ABA. We provide physiological and molecular evidence that ethylene and ABA regulate pepper fruit ripening differently in a process distinct from the climacteric and non-climacteric types of ripening: (1) a peak of ethylene emission was observed at the IC stage, followed by a peak in the RQ at the Br stage and rapid accumulation of ABA after the Br stage, which reached a maximum level at the FR stage (Fig. 1); (2) ethylene promoted ripening, ABA played no role in ripening, and fluridone accelerated ripening (Fig. 2); (3) the most abundant phytohormone-related DEGs were related to ABA (10 genes) and ethylene (6 genes), and no more than two DEGs related to other phytohormones were identified (Fig. 4); especially higher expression was observed for *ACO3*, which is key to ethylene biosynthesis, and for *NCED1*/*3*, which are key to ABA biosynthesis (Fig. 5); and (4) the downregulated expression of *NCED1*/*3* and *ACO3* promoted and inhibited colouration, respectively, consistent with the results of our physiological and molecular analyses (Figs. 5, 6).

To our surprise, exogenous ABA played no role in colouration, but fluridone did promote colouration (Fig. 2). This phenomenon was also confirmed by the downregulation of *NCED1/3* expression by VIGS (Fig. 5). Similarly, a significant reduction in NCED activity leading to carotenoid accumulation was previously reported in tomato fruit [51]. Indeed, pepper fruit colours result from the composition and amount of diverse carotenoids, which are synthesised by a set of genes including *PSY*, *PDS*, *ZDS*, *BCH*, *ZEP*, and *CCS* [40, 42, 52, 53], consistent with our RNA-seq data (Fig. 4). To some extent, the ZEP/ABA1 screen is a common substrate for carotenoids and ABA biosynthesis, which may reflect a complex role for ABA in hot pepper fruit ripening.

Two early, sequential committed steps in the conversion of farnesyl pyrophosphate (FPP) to phytoene in carotenoid biosynthesis are catalysed by geranylgeranyl diphosphate synthase and phytoene synthases [54]. Notably, the recent reports show that squalene synthase (SQS) is involved in carotenoid biosynthesis, and can convert to a dehydrosqualene synthase for production of the C_30_ carotenoid backbone [55, 56]. An early report showed the conversion of FPP to squalene by SQS in hot pepper [57]. In this study, the transcriptome data showed that *SQS* expression levels, on the whole, exhibited a continually decreasing trend at the onset of ripening (Additional file 1: Figure S2a). Fluridone can inhibit PDS activity [58, 59]. We showed that fluridone led to an increase in *SQS* transcript levels in fluridone-treated fruits (Additional file 1: Figure S2b), finally initiated the ‘squalene route’ for carotenoid biosynthesis [56]. To a large extent, the colouration of pepper fruit promoted by fluridone treatment resulted from the upstream accumulation of C_30_ carotenoid once PDS activity is inhibited by fluridone.

Paradoxically, although endogenous ABA levels increased rapidly after the Br stage (Fig. 1), exogenous ABA did not affect colouration (Fig. 2). Concurrent with ABA accumulation during colouration, the transcript levels of *SnRK2*, a positive regulator of ABA signalling in the ABA‘PYR-PP2C-SnRK2’ core signalling pathway [26], declined continuously, while the transcript levels of *PP2C*, a negative regulator of ABA signalling, increased continuously (Figs. 1, 4) and inhibited ABA signalling at the transcriptional level. In contrast, concurrent with a decrease in ethylene emission during colouration, the expression of ethylene signalling-related DEGs, including *CTR1*, *CTR2*, *EIN2*, *EIN3*, and *ERF1*, promoted ethylene signalling (Figs. 1, 4). In conclusion, a peak in ethylene emission resulting from high *ACO3* mRNA expression levels at the IC stage triggered the hot pepper fruit ripening through a coordinated expression pattern of ethylene-signalling genes during colouration (Figs. 1, 4). Although a rapid increase in ABA content was consistent with high *NCED1* mRNA expression levels during fruit colouration, ABA signalling was weakened by the uncoordinated expression of ABA-signalling genes (Figs. 1, 4). Ethylene and ABA regulate hot pepper fruit ripening differently, with ethylene promotes colouration while ABA might play a role in de-greening. Our results provide new insights into understanding of fleshy fruit ripening.

## Conclusions

We have provided a series of pharmacological, physiological and molecular evidences to demonstrate that during hot pepper (*C. frutescens*) fruit ripening, higher *ACO3* expression levels are associated with an ethylene emission peak, which triggers the onset of ripening, demonstrating that ethylene acts as a positive regulator of the ripening. The higher *NCED1*/*3* expression levels are coupled with ABA accumulation during ripening. Exogenous ABA does not promote colouration, and inhibition of ABA biosynthesis accelerates colouration. Our findings provide new insights into processes of fleshy fruit ripening regulated by ABA and ethylene, focusing on ethylene in carotenoid biosynthesis and ABA in chlorophyll degradation.

## Methods

### Plant material

Red, clustered, upright, hot pepper (*C. frutescens* ‘Chaotianjiao 6’) plants were grown in a greenhouse (25–35 °C, relative humidity 70–90%, 12/12-h light/dark regime) in 2015 and 2016. Four hundred flowers on 50 plants were tagged during flowering. Developmental fruits were divided into six stages (YG, SG, LG, IC, Br, and FR) at 1, 2, 3, 4, 5, and 6 weeks after anthesis, respectively.

### Determination of ethylene production

Ten uniform pepper fruits attached to the plants at each stage were sampled and placed in 1-L glass jars for 1 h at 25 °C. One millilitre of the jar headspace gas was extracted and injected into a gas chromatograph equipped with a flame ionisation detector and an activated alumina column (model 6890 N; Agilent Technologies, USA). Ethylene production (μL hr.^− 1^ kg ^− 1^ FW) was analysed by column chromatography, including 5% phenyl methyl siloxane and a 30-m capillary alumina column (19,091 J-413; Agilent Technologies). The temperatures of the column and detector were 80 °C and 150 °C, respectively. The rate of N^2^ carrier gas flow was 40 mL min^− 1^, and the hydrogen pressure was 0.6 kg cm^− 2^. The experiment was repeated three times.

### Determination of ABA content

Three frozen pepper fruits of each ripening stage were selected randomly for determination of ABA content by gas chromatography-mass spectroscopy (GC-MS). One gram of pericarp tissue was mixed with antioxidant copper reagent, quartz sand, a small amount of 80% cold methanol, and D_3_-ABA (an internal standard). The mixture was ground and mixed with 80% methanol, then soaked overnight at 4 °C for 12 h. Filtration, supernatant collection, extraction with a C_18_ column, and GC-MS analysis were performed as described previously [25]. The experiment was repeated three times.

### Determination of carotenoid and chlorophyll contents

Three uniform pepper fruits of each ripening stage were used to determine carotenoid and chlorophyll contents by high-performance liquid chromatography (HPLC) using 5 g pericarp tissue. ß-Carotene, zeaxanthin, and capsanthin were purchased from Sigma-Aldrich (Munich, Germany). A Sep-Pak Silica 6-cc Vac Cartridge solid-phase extraction column (Waters, Milford, MA, USA) was used. Acetonitrile, methanol, and methyl *tert*-butyl ether were HPLC grade. Chromatographic analysis was performed using an Agilent 1260 HPLC system. An analytical C30 column (250 × 4.6 mm, 5 μm; YMC Co., Japan) was used. Separation was carried out using methanol/*tert*-butyl methyl ether (70:30, *v*/v) at a flow rate of 1.0 mL/min with a column temperature of 25°C. Chromatograms were monitored at a UV wavelength of 450 nm. The procedures were carried out as described previously [40]. Chlorophyll contents were determined as described previously [60]. The experiments were repeated three times.

### Determination of soluble sugar and starch contents

Three uniform pepper fruits of each ripening stage were used to determine the sugar contents of pericarp tissue (1 g) using reverse-phase HPLC (RID1 A detector, 1200 series; Agilent Technologies). The supernatant was fractionated using a Sugar-PakTM1 column (6.5 × 300 mm, Waters) with 100% MilliQ water for 25 min at the flow rate of 0.4 mL per min. The column temperature was 80°C and the injection volume was 20 μL. Standard samples used were D-(+) glucose, D-(−) fructose, and sucrose (Sigma-Aldrich). The HPLC analysis was done as described previously [25]. Starch contents were detected using a total starch assay kit (Megazyme International Ireland Ltd., Wicklow, Ireland) according to the manufacturer’s protocol. The experiments were repeated three times.

### Determination of RQ

Three uniform pepper fruits of each ripening stage were used to determine the RQ of pericarp tissue (5 g) using a Warburg microrespirometer. Absorption of oxygen was determined with the addition of 20% NaOH solution to the reaction bottle, and the release of carbon dioxide was determined with the removal of 20% NaOH solution. The RQ was the ratio of the amount of carbon dioxide released to the amount of oxygen absorbed. The experiment was repeated three times.

### Effects of ABA, ethylene, and inhibitors on pepper fruit ripening

Seven LG fruits attached to plants were selected and immersed for 2 min in 50 μM ABA, 100 μM fluridone, 50 μM ethephon (2-chloroethylphosphonic acid, an ethylene-releasing agent), or 1 mL/L 1-MCP (an inhibitor of ethylene release), as described previously [61, 62], with immersion in water used as a control. The treated and control fruits were placed in a growth chamber (25 °C, relative humidity 70–80%, 12/12-h light/dark regime) for 8 days. The experiment was repeated three times.

### RNA extraction, library construction, and RNA-seq analysis

RNAs were extracted from pericarp tissues from four uniform pepper fruits of each ripening stage, including LG, IC, Br, and FR, using the RNeasy Plant Mini Kit (Qiagen, Dusseldorf, Germany) for RNA-seq analysis. DNase digestion by RNase-Free DNase (Qiagen) removed contaminating DNA, and RNA samples were processed sequentially using an RNA Library Prep Kit (New England BioLabs, Ipswich, MA, USA). RNA-seq and data analysis were performed on the Illumina HiSeq2000 (Illumina, San Diego, CA, USA) by the Beijing Yuanyi Gene Science Company (Beijing, China). Unigenes with adjusted *P*-values < 0.05 and absolute values of log_2_ (expression fold change) > 1 were deemed to be differentially expressed, and false discovery rate-adjusted *P*-values < 0.05 for unigenes were considered to be statistically significant [63–67]. The experiment was repeated three times.

### VIGS

The pTRV1 and pTRV2 vectors were kindly provided by Dr. Yu-le Liu (Qinghua University, Beijing, China). On the basis of the RNA-seq data, primers with an *Xba*I restriction site in the forward primer and a *Kpn*1 restriction site in the reverse primer (underlined below) were designed for amplification of the partial gene sequences of *ACO3* (GenBank accession no. NM_001324767), *NCED1* (GenBank accession no. XM_016697078), and *NCED3* (GenBank accession no. XM_016691094). The primers used were as follows: a 452-bp cDNA fragment of *ACO3* (forward, 5´-GCTCTAGAAAAAGGGCTGAAACAATGGAC-3′ and reverse, 5´-GGGGTACCGGACAAGGAGGGTAG TTGCTAA-3′), a 537-bp cDNA fragment of *NCED1* (forward, 5´-GCTCTAGAAATCTCTACAAGTGACCGGA-3′ and reverse, 5´-GGGGTACCGAATCACATCATAGCTAAGAG-3′), and a 600-bp cDNA fragment of *NCED3* (forward, 5´-GCTCTAGACAGTGAGTTATTCTTGCCGTT-3′ and reverse, 5´-GGGGTACCACCTGGATTTCTTCTTTTTG TC-3′). These amplified fragments were inserted into the pEASY-T1 Simple Cloning Kit (TransGen Biotech, Beijing, China), and then into the TRV pTRV2 vector between the *Xba*I and *Kpn*1 sites. The *Agrobacterium tumefaciens* strain GV3101 containing pTRV1, pTRV2, and pTRV2-ACO3/NCED1/NCED3 was used for VIGS. Cell suspension of each TRV2 vector carrying target genes was mixed with TRV1 cell suspension at a 1:1 (*v*/v) ratio, and 0.2-mL volumes were infiltrated into seven LG fruits as described previously [40]. Empty TRV vector was used as a control. After inoculation, the fruits were covered with black paper for 24 h. The experiment was repeated three times.

### qPCR

The relative expression levels of carotenoid synthesis genes in transgenic fruits were determined using semi-quantitative reverse-transcription PCR. Three transgenic fruits were sampled for RNA isolation and cDNA synthesis as described above using pericarp tissue. First-strand cDNAs corresponding to the genes were used as templates during PCR amplification through 24 or 28 cycles. Expression of the *Ubi3* gene was used as an internal reference [40]. The primers for qPCR are shown in Additional file 1: Table S1. The experiment was performed with three replications.

## Additional files

Additional file 1: **Table S1.** The primers for qPCR based on RNA-Seq data. **Figure S1.** Phenotype of the red, cluster, upright and hot pepper plant (*Capsicum frutescens*, cv. ‘Chaotianjiao 6’). **Figure S2.** Fluridone-promotion of the fruit coloration is related to squalene synthase (SQS) expression at transcription levels. (DOC 4362 kb)

## Abbreviations

AAO: Abscisic-aldehyde oxidase
ABA: Abscisic acd
ABI: Abscisic acid-insensitive
ACO: 1-aminocyclopropane-1-carboxylate oxidase
ARR: Two-component response regulator
BCH/crtZ: Beta-carotene hydroxylase
CCS/lcyB/crtY: Capsanthin/capsorubin synthase
CTR: Ethylene response sensor
CYP707: Abscisic acid 8′-hydroxylase
EIN: Ethylene-insensitive protein
ERF: Ethylene-responsive transcription factor
GH3: Indole-3-acetic acid-amido synthetase
GID1: Gibberellin receptor
IAA: Auxin-responsive protein
MYC2: Myelocytomatosis transcription factor
NCED: 9-cis-epoxycarotenoid dioxygenase
PDS/crtP: 15-cis-phytoene desaturase
PP2C: Protein phosphatase 2C
PSY/crtB: 15-cis-phytoene synthase
PYL: Abscisic acid receptor
SnRK: Serine/threonine-protein kinase
ZDS/crtQ: Zeta-carotene desaturase
ZEP/ABA: Zeaxanthin epoxidase
