# Supplementary material for: Characterization of the hot pepper (Capsicum frutescens) fruit ripening regulated by ethylene and ABA
Source: BMC Plant Biol. 2018 Aug 10;18:162. doi: 10.1186/s12870-018-1377-3 (PMC6086059; doi:10.1186/s12870-018-1377-3)
Supplement: Supplementary file 1 — Table S1. The primers for qPCR based on RNA-Seq data. Figure S1. Phenotype of the red, cluster, upright and hot pepper plant (Capsicum frutescens, cv. ‘Chaotianjiao 6’). Figure S2. Fluridone-promotion of the fruit coloration is related to squalene synthase (SQS) expression at transcription levels. (DOC 4362 kb) [file 12870_2018_1377_MOESM1_ESM.doc]

**Additional files**

**Additional file 1: Table S1.** The primers for SqRT-PCR based on RNA-Seq data.

| Pepper genes | Primer sequences |
| --- | --- |
| *CCS* | Sense: 5'- TGGCTATTGGTGGGACT -3';  Antisense: 5'- GAAGAAAGGAACCCGTG-3'. |
| *PSY* | Sense: 5'- TCCCGCTGAATACCTGG -3';  Antisense: 5'- GTCGTGATAAACCTGCCTTG -3'. |
| *PDS* | Sense: 5'- AATGCCAAACAAGCCAGGA -3';  Antisense: 5'- GAGTCGCAAACACAAAAGC -3'. |
| *ZEP* | Sense: 5'- GTTTGCCGATGACCACA -3';  Antisense: 5'- CAAGAACGGTATCAGGAGAG -3'. |
| *ZDS* | Sense: 5'- GGGGGAAGGTTTCATCT -3';  Antisense: 5'- CCAGCCATTGTATCGTAGT -3'. |
| *BCH* | Sense: 5'- TCATTCTCGTCCACCTCC -3';  Antisense: 5'- GCAAAAACATCGTTCAGC-3'. |
| | *Ubi3* | | --- | | | Sense, 5'- GTTGTCTCTCGTCTACCTGT-3';  antisense, 5'- CAGAATAACACGAACCCCAC -3' | | --- | |


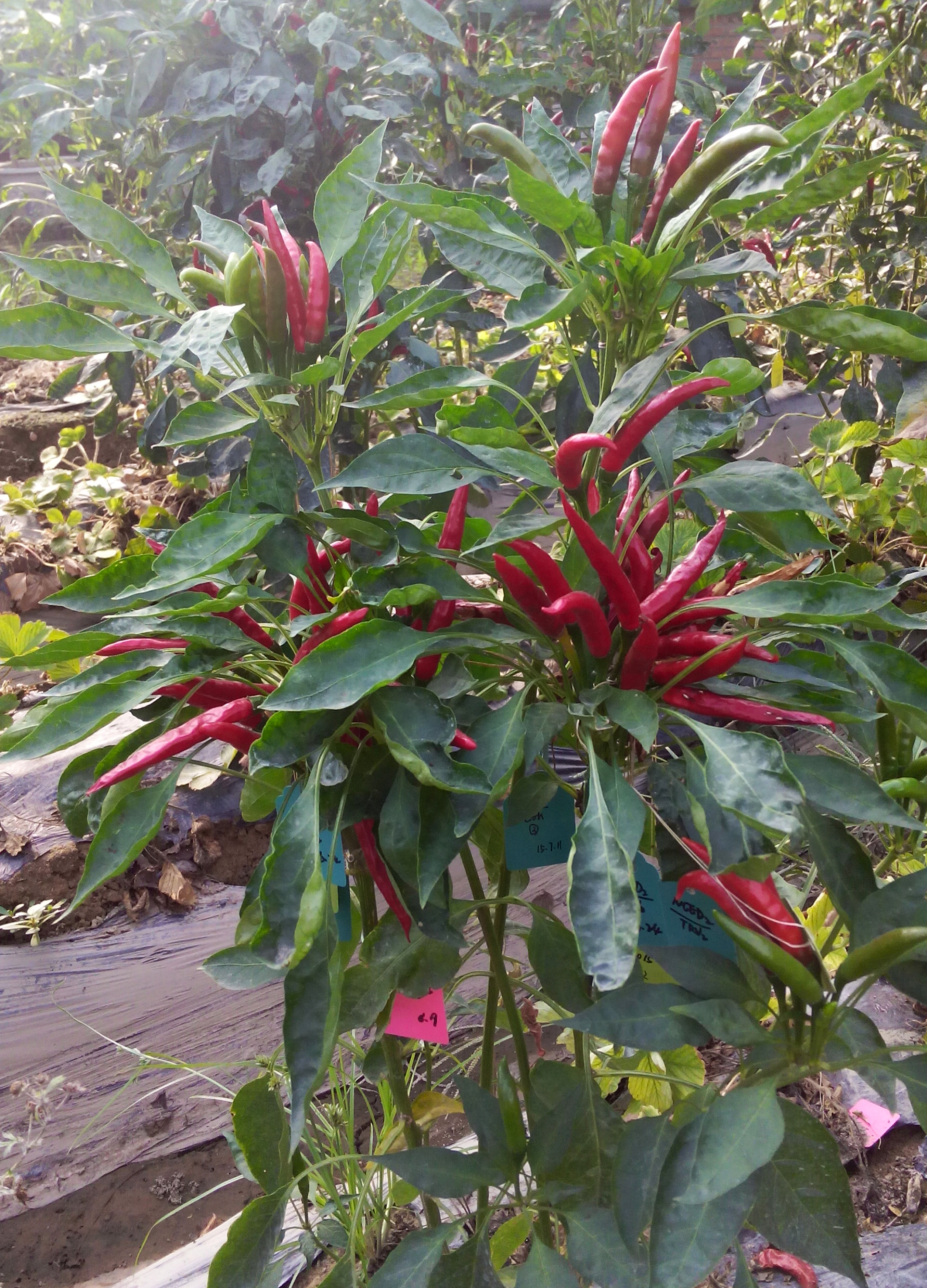


**Additional file 2: Figure S1.** Phenotype of the red, cluster, upright and hot pepper plant (*Capsicum frutescens*, cv. ‘Chaotianjiao 6’).


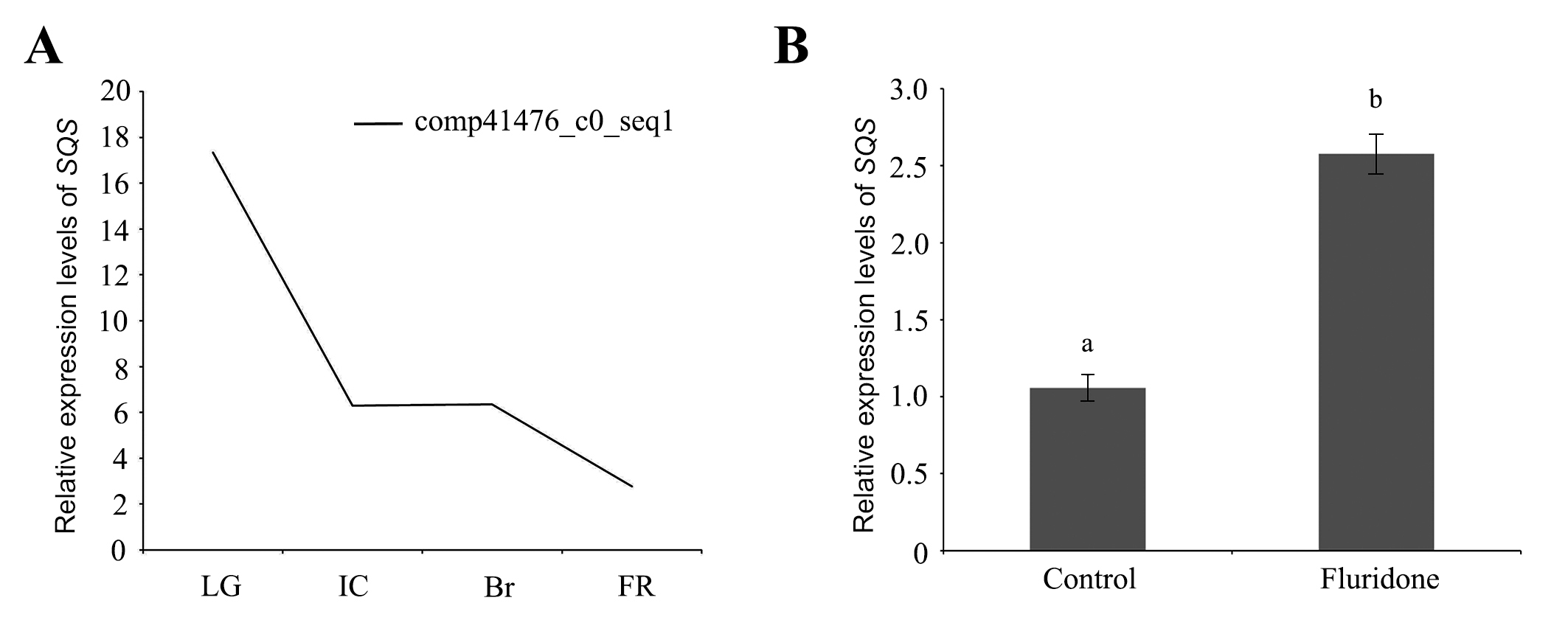


**Additional file 2: Figure S3.** Fluridone-promotion of the fruit coloration is related to squalene synthase (SQS) expression. (a) RNA-Seq analysis of SQS expression during the fruit ripening. LG: large green); IC: initial coloring; Br: brown; FR: full red. (b) Fluridone promoted SQS transcripts. *Ubi3* was used as the internal control. Error bars represent the SE (SEs; *n* = 3). Columns with different letters (a, b) indicate statistically significant difference (p < 0.05) when the data are performed by variance analysis followed by Duncan’s multiple range tests.
